# Vaccination

**Published:** 1900-03

**Authors:** H. A. Engelhardt

**Affiliations:** Houston, Texas; Former House Physician at the Almshouse and Workhouse Hospitals, B. I., New York City


For Texas Medical Journal.

### Vaccination.

BY DR. H. A. ENGELHARDT, HOUSTON, TEXAS.
Former House Physician at the Almshouse and Workhouse Hospitals, B. I., New York City.

Smallpox or variola has been without doubt since Christ until the beginning of this century the most frequent, the most widespread, and the most deadly disease. Its existence in Asia, where it probably originated, was many centuries before Christ.

In Europe the disease has reigned since the beginning of the sixteenth century, and shown itself during that time in various epidemics and pandemics throughout the country. Wherever commerce went, with it followed smallpox, even to the most far-off new discovered countries.

The mortality among aboriginal people, who enjoyed previous immunity, is very high. With the introduction of variola into America the Mexicans died by the thousands, and the North American Indians have also been frequently decimated by this plague.

The mortality in children through smallpox has to some extent had influence on the European population. The most frequent epidemics in Europe occurred in the eighteenth century, particularly in the latter part. In the beginning of the nineteenth century the death rate decreased, due to the introduction of vaccination. The new discovery, which Dr. Jenner published in London, in 1798, was received with great enthusiasm throughout the world, already in 1802 the number of persons vaccinated in England amounted to more than 100,000. Vaccination now spread rapidly over her colonies and the rest of Europe. From Spain it was brought to Spanish America in 1803. In 1817 another epidemic occurred in Scotland and spread over England and the continent. During this epidemic varioloid was recognized for the first time. The smallpox epidemic in 1830-34, and 1840-50, and particularly the last and longest epidemic in 1869-73, was due to the many opponents to vaccination, and partially to the distastrous results of vaccination in the hands of incompetent persons. The loss of life which occurred during the last epidemic was, in Germany, and more so in France, by far larger than the losses in the Franco-German war.

In consequence of this calamitous smallpox epidemic, the German Empire enacted the law of 1874, which makes vaccination obligatory in the first year of life, and revaccination obligatory at the 10-12 year. What was the result? With a population of 50,000,000, having in 1871 lost 143,000 lives by smallpox, she found by her loss of 1874 the mortality diminished so rapidly that today the disease numbers only 116 victims a year; these cases, moreover, occur almost exclusively on her frontier.

Laws of compulsory vaccination were in force long before this. Bavaria already in 1807; Sweden, 1810; Baden, 1815; Hanover, 1821; Saxony, 1828; and Prussia, 1835. Experience soon taught that one successful vaccination did not give immunity for life time, and it made it necessary to repeat vaccination. Revaccination was introduced in the Prussian army in 1834, and the result was as follows:

| | | |
|---|---|---|
| Before vaccination, | 1825-34 | 458 deaths. |
| After vaccination, | 1835-44 | 41 deaths. |
| | 1845-54 | 13 deaths. |
| | 1855-64 | 12 deaths. |

In Vienna, where revaccination was not practiced, the death rate in the years 1875-83 was 89.3 to 100,000 inhabitants, while in Berlin, where revaccination was enforced, it was only 1.61 per cent.

Dr. Davis P. Austin, of New York, who has had a wide experience in vaccination, favors vaccination every year, until immunity is secured; ordinarily this immunity could be produced in a child at the end of five years, and if used in this way, they would be immune for the rest of their lives.

Does vaccination protect from smallpox? Yes; if it did not, we ought to find in smallpox epidemics that the disease diffuses itself in the well vaccinated no less than in the non-vaccinated countries. But this is not so. During the Franco-German war, the two peoples intercommingled with each other, the German having its civil population vaccinated optionally, but the army completely vaccinated, while the French population and army were vaccinated perfunctory. Both were attacked by smallpox, but the French army numbered 23,000 deaths by it, while the German army had only 278, and in the same tent, breathing the same air, the French wounded were heavily visited by the disease, while the German wounded, having been vaccinated, had not a single case.

When does immunity begin, and how long does it last? The beginning of immunity after a successful vaccination occurs usually at the eighth to ninth day, the duration of immunity varies, as a rule eight to ten years as given by most authors. A clinical fact is that the length of immunity against natural infection is longer than that against vaccine. Statistics also show that after a successful vaccination the duration of the immunity was very much longer, although not likely to last the rest of one's life. Having 15,000 cases on record, the deaths from smallpox among vaccinated persons had been 35 per cent.; in those of one vaccination it had been 7.73 per cent.; in those with two scars 4.7 per cent.; and in those with three vaccinations 1.95 per cent.; while in those with four or more scars of vaccination it had been 0.55 per cent.; hence when there were several scars indicating successful vaccinations, the mortality from smallpox was comparatively less. An experienced physician of health department said, that it was much easier to vaccinate a person than it was to contract the disease after successful vaccination.

### NATURAL IMMUNITY.

Cases have been reported of this kind, but the greater one's experience is in vaccination, the more skeptical one becomes on this point. Dr. Austin cites a case in which a boy fourteen years old, who had been repeatedly vaccinated without success, and who had finally been successfully vaccinated when human lymph had been employed.

### SCARS AN UNTRUSTWORTHY GUIDE.

Vaccination scars absolutely signify nothing, as has been repeatedly proven clinically, that poor scars were quite resistant to vaccination and vice versa.

To be absolutely certain of immunity is to be vaccinated again and again.

### TIME OF VACCINATION.

The time at which vaccination should be done, I think of considerable importance. Any time of the year is acceptable, except the hot period during the summer months, as the heat influences the course of vaccination and also complications are more ready to appear. The best time to vaccinate an infant is when it is from four to six months old, that is the time before the first tooth appears. The advantage is also, that at this time the child has become accustomed to its food, its digestion is in equilibrium and its power to resist (widerstandsfähigkeit) is so much greater than at other times.

Dr. Rotch mentions a valuable point, that by the fifth month it will usually have developed the outward symptoms of syphilis if it has inherited that disease from its parents, you will then not be as likely to be blamed for having innoculated with something besides vaccine virus, which might happen if after vaccination in early weeks of life a syphilistic effervescence should appear. Should it be necessary to vaccinate an infant in the first weeks of life, it should be done with particular care and asepsis and with only few scratches.

A few words in regard to the opponents of vaccination. Anti-vaccinators have pointed to the danger from infection, as with syphilis, tuberculosis, scrofula, rachitis, and many others; also they claim, that through vaccination the constitution of the whole population suffers, that it makes one more susceptible to other diseases, particularly to typhoid, dysentery, scarlet fever, and measles, and above all, they vouch that through it the mortality is increased.

As regards to the latter opposition, I have already said enough, but will say a few words on syphilis and tuberculosis. With the exception of syphilis not one of the other diseases has any relation to vaccination. A few, a very few, isolated cases of syphilis have undoubtedly occurred, but the introduction of bovine virus has now done away with this great objection. Tuberculosis has never been noted. In the virus of tubercular individuals there were never found tubercular bacilli, and experiments to infect guinea pigs with the virus of far advanced cases of tuberculosis and also those from calves, which had far advanced with the disease, has never been accomplished.

In conclusion, vaccination and revaccination do not only guard the individual vaccinated against the danger and severity of the disease, but the general introduction in all classes of people will, through the prevention from contagion and infection, increase in a very high degree the security to the whole population.

